# Combined use of pritelivir with acyclovir or foscarnet suppresses evolution of HSV-1 drug resistance

**DOI:** 10.1093/ve/veae101

**Published:** 2024-11-23

**Authors:** Hanna Helena Schalkwijk, Graciela Andrei, Robert Snoeck

**Affiliations:** Laboratory of Virology and Chemotherapy, Department of Microbiology, Immunology and Transplantation, Rega Institute for Medical Research, KU Leuven, Herestraat 49 box 1043, Leuven 3000, Belgium; Laboratory of Virology and Chemotherapy, Department of Microbiology, Immunology and Transplantation, Rega Institute for Medical Research, KU Leuven, Herestraat 49 box 1043, Leuven 3000, Belgium; Laboratory of Virology and Chemotherapy, Department of Microbiology, Immunology and Transplantation, Rega Institute for Medical Research, KU Leuven, Herestraat 49 box 1043, Leuven 3000, Belgium

**Keywords:** herpes simplex virus 1, antiviral resistance, combination therapy

## Abstract

The widespread use of antivirals in immunocompromised individuals has led to frequent occurrences of drug-resistant herpes simplex virus 1 (HSV-1) infections. Current antivirals target the viral DNA polymerase (DP), resulting in cross-resistance patterns that emphasize the need for novel treatment strategies. In this study, we assessed whether combining antivirals with different targets affects drug resistance emergence by passaging wild-type HSV-1 under increasing concentrations of acyclovir (ACV), foscarnet (phosphonoformic acid, PFA), or the helicase–primase inhibitor pritelivir (PTV), individually or in combination (ACV + PTV or PFA + PTV). The resistance selection procedure was initiated from two different drug concentrations for each condition. Deep sequencing and subsequent phenotyping showed the rapid acquisition of resistance mutations under monotherapy pressure, whereas combination therapy resulted in either no mutations or mutations conferring ACV and/or PFA resistance. Notably, mutations associated with PTV resistance were not detected after five passages under combination pressure. Strains resistant to both ACV and PTV were eventually obtained upon further passaging under ACV + PTV pressure initiated from lower drug concentrations. PFA + PTV dual treatment induced PFA resistance mutations in the DP, but PTV resistance mutations were not acquired, even after 15 passages. Our data suggest that combining the helicase–primase inhibitor PTV with a DP inhibitor may be an effective strategy to prevent drug resistance evolution in HSV-1.

## Introduction

The treatment of herpes simplex virus 1 (HSV-1) infections to date has relied on inhibition of the viral DNA polymerase (DP). The nucleoside analog acyclovir (ACV) and its prodrug valacyclovir are the first-line treatment for HSV-1 infections. Apart from nucleoside analogues, which require viral thymidine kinase (TK) activity to become activated (Deville-Bonne et al. 2010), other DP inhibitors have been developed. These include foscarnet (PFA), which directly targets the DP pyrophosphate binding site, and cidofovir (CDV), which is independent of the viral TK for activation and acts as an alternative DP substrate following activation by cellular kinases (Schalkwijk et al. 2022b).

Unfortunately, the available anti-HSV-1 drugs are burdened by drug resistance, particularly in immunocompromised hosts and during infections of immune-privileged sites such as the cornea (Schalkwijk et al. 2022b). ACV resistance is predominantly associated with TK mutations and to a lesser extent with DP mutations, which often cause resistance to both ACV and PFA (Piret and Boivin 2011). Foscarnet has been approved for the treatment of ACV-resistant HSV-1 infections due to viral TK alterations in immunocompromised individuals, whereas CDV has been used off-label to treat HSV-1 infections unresponsive to ACV and/or PFA (Piret and Boivin 2021). (Multi)drug-resistant HSV-1 infections form a therapeutic challenge, emphasizing the need for new treatment strategies (Anton-Vazquez et al. 2020, Schalkwijk et al. 2023).

A new class of antivirals under investigation targets the viral helicase–primase complex, which is essential for unwinding and priming viral DNA prior to DNA synthesis (Piret and Boivin 2021). Two helicase–primase inhibitors, pritelivir (PTV) and amenamevir (AMV), are under clinical evaluation for the treatment of HSV infections (i.e. HSV-1 and HSV-2). AMV is licensed in Japan for the therapy of herpes zoster, caused by varicella-zoster virus reactivation, and since 2023, it is additionally approved for recurrent herpes simplex infections (Kawashima et al. 2022, Kawamura et al. 2024). PTV solely exhibits anti-HSV activity, and a phase 3 trial for the treatment of ACV-resistant HSV infections in immunocompromised subjects is ongoing (NCT03073967). Multiple immunocompromised patients suffering from refractory HSV infections were treated successfully with PTV via an early access program (Cannon et al. 2021, Serris et al. 2022, Bosetti et al. 2023, Huntjens et al. 2023).

A potent way to prevent drug resistance emergence is the usage of multiple drugs simultaneously, particularly drugs with distinct modes of action. Combinations of two or more drugs are used to treat various infectious diseases as well as cancer but are only sporadically applied to treat HSV infections (Pirrone et al. 2011, Schalkwijk, et al., 2022b). Several DP inhibitors and helicase–primase inhibitors—ACV combined with AMV or PTV, and CDV with AMV—were found to have additive or synergistic effects on HSV-1 replication (Chono et al. 2013, Quenelle et al. 2018, Greeley et al. 2020). However, data on their combined effect on drug resistance emergence are lacking.

In this study, we analyzed the resistance profile of HSV-1 strains emerging under pressure of two antivirals—ACV or PFA in combination with PTV—compared to HSV-1 strains selected under single drug pressure.

## Materials and methods

### Cells and viruses

Human embryonic lung (HEL) fibroblasts (ATCC CCL-137) were propagated in Dulbecco’s modified Eagle’s medium (DMEM) with 8% fetal bovine serum (FBS), 0.1 mM non-essential amino acids, 1 mM sodium pyruvate, 2 mM l-glutamine, and 10 mM N-2-hydroxyethylpiperazine-N-2-ethane sulfonic acid (HEPES) at 37°C in a 5% CO_2_ humidified atmosphere. The HSV-1 wild-type strain KOS (ATCC VR-1493) was propagated in HEL fibroblasts (DMEM, 2% FBS).

### Compounds

The sources of compounds are as follows: ACV (Cat. No. PHR1254) and foscarnet (PFA, Cat. No. P6801) (Merck), adefovir (9-(2-Phosphonylmethoxyethyl)adenine, PMEA) and CDV (kindly provided by Gilead Sciences), AMV (Cat. No. 27921-5) (Cayman Chemical), ganciclovir (GCV, cymevene) (Roche), PTV (Cat. No. HY-15303) (MedChemExpress), and trifluridine (TFT, Cat. No. 4460) (Tocris Bioscience).

### Synergistic activity

The combined activity of PTV with ACV or PFA against HSV-1 was determined using virus yield inhibition assays as previously described (Schalkwijk et al. 2024). Dose–response matrixes and 2D synergy maps were generated in SynergyFinder 2.0 (https://synergyfinder.fimm.fi) using the zero interaction potency (ZIP) model. The ZIP model quantifies the degree of synergy of the tested drug combinations assuming that the single drugs do not affect the potency of each other (Ianevski et al. 2020). The divergence from the expected response for each dose pair was quantified by calculating synergy (*δ*) scores, with a *δ* score < −10 implying antagonistic, a score between −10 and 10 additive, and a score >10 synergistic interactions.

### Cytotoxicity assays

Cytostatic effects of the antiviral compounds individually (ACV, PFA, or PTV) and combined (ACV + PTV or PFA + PTV) on HEL fibroblasts were measured using cell growth inhibition assays as described previously (Schalkwijk et al. 2024). SyngeryFinder 2.0 software was used to determine potential synergistic effects.

### Drug resistance selection

The KOS strain was sequentially passaged in HEL fibroblasts under increasing concentrations of one (PFA and PTV) or two (ACV + PTV or PFA + PTV) compounds as described previously (Schalkwijk et al. 2024). Drug resistance selection under ACV pressure has been described in a former publication (Schalkwijk et al. 2024). For each condition, resistance selection was initiated from two different drug concentrations. The initial and final drug concentrations used during the resistance selection are available in Supplementary Table S1. Briefly, cell cultures were incubated until the development of full virus cytopathic effect (CPE) was observed. The viruses were harvested by freeze-thawing and used to infect fresh cell cultures, increasing the concentrations two-fold in each subsequent passage. If full CPE was not obtained within 10 days postinfection, the medium was refreshed with drug-free medium every 5 days until full CPE was obtained, followed by a passage at the same drug concentrations. After five passages, the virus cultures were genotyped by Sanger and deep sequencing. Viral clones were plaque-purified from the virus cultures and genotyped by Sanger sequencing.

### Sequencing

The QIAmp DNA blood kit (Qiagen) was used to extract viral DNA from the virus cultures and viral clones. Sanger sequencing of the *UL23* (TK), *UL30* (DP), *UL5* (helicase), and *UL52* (primase) genes was performed as previously described (Schalkwijk et al. 2022a).

Amplicon-based next-generation sequencing (NGS) was used to determine the frequency of mutations in the virus cultures after two and five passages. The full TK and *UL52* genes were amplified by polymerase chain reaction (PCR) (Platinum SuperFi, Invitrogen), while partial amplification of the DP and *UL5* genes, encompassing the regions known to confer drug resistance, was carried out (Supplementary Table S2). Amplicons were sequenced on the MiSeq v2 system as described previously (Andrei et al. 2019). Reads were mapped to the HSV-1 strain 17 reference genome (GenBank accession number NC_001806.2) using CLC genomics workbench 12 (Qiagen). Variants with a frequency of >1% were called using the low-frequency variant detection tool. The raw sequencing data were submitted to the GenBank Sequence Read Archive (SRA) database under BioProject PRJNA1119278.

### CPE reduction assays

HEL fibroblasts were inoculated with 100-fold the 50% cell culture infective dose of various virus clones, and serial dilutions of compounds were added 2 hours postinfection. CPE was scored 72 h postinfection from which EC_50_ (50% effective concentration) values were calculated. Viral clones were considered resistant at fold resistance (EC_50_ mutant/EC_50_ wild-type) values ≥2.

## Results

### Synergistic activity of PTV in combination with ACV or PFA

**Figure 1.**
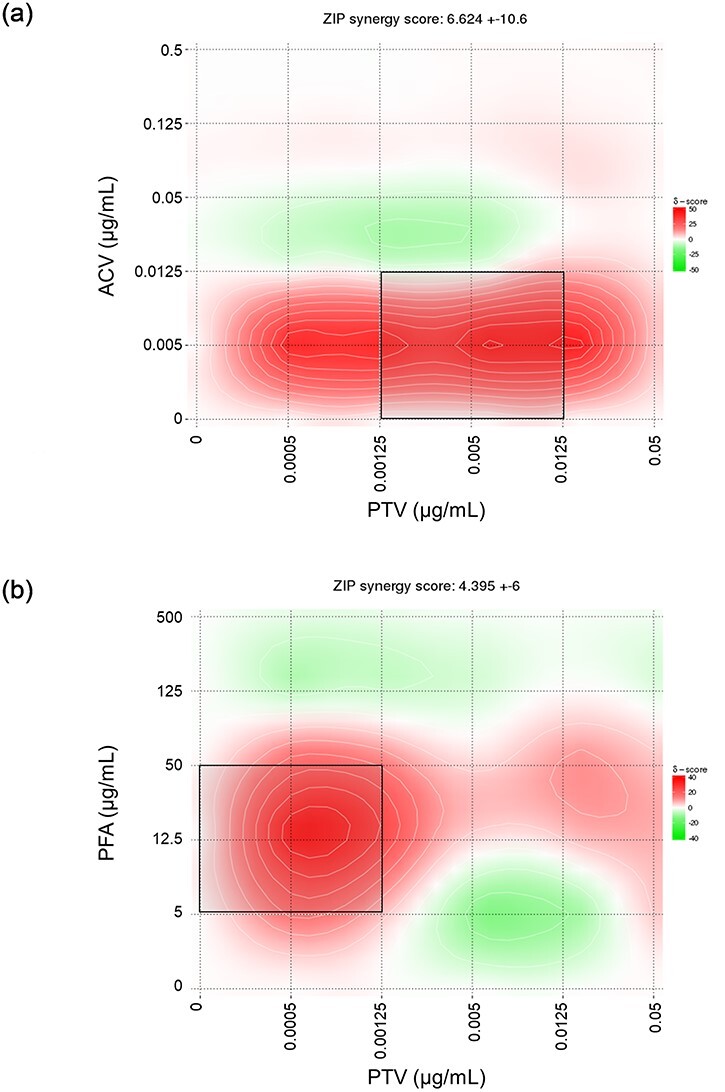
Anti-HSV-1 activity of PTV in combination with ACV or PFA. HEL fibroblasts infected with HSV-1 wild-type strain KOS were exposed to various concentrations of one or two antivirals (*n* = 2). Inhibitory effects of the antivirals were assessed by determining the reduction in HSV-1 virus titers. Interaction plots for (a) ACV and PTV and (b) PFA and PTV were generated using SynergyFinder 2.0 using the ZIP model. Synergy (*δ*) scores were calculated to quantify the deviation from the expected response for the given dose pair, with a *δ* score < −10 implying antagonism, a *δ* score between −10 and 10 implying additivity, and a *δ* score >10 implying synergism. Overall synergy scores, based on all measured drug combinations, are shown above the plots, and the black squares indicate the most synergistic 3-by-3 dose region in the dose–response matrix.

The inhibitory effects of PTV in combination with ACV or PFA on HSV-1 were evaluated using virus yield inhibition assays, and the obtained dose–response matrixes were analyzed using SynergyFinder 2.0 (Fig. 1). The average *δ* score, calculated for the entire dose–response matrix, revealed additive effects for the ACV + PTV (6.624 ± 10.6) and PFA + PTV (4.395 ± 6) combinations, whereas *δ* scores calculated for the most synergistic 3-by-3 dose region in the dose–response matrix (Fig. 1, black box) implied synergy for both ACV and PTV (13.54) and PFA and PTV (12.14). The observed synergistic effects on HSV-1 inhibition were not associated with an increase in cytotoxicity (Supplementary Fig. S1).

### Sequence analysis of HSV-1 cultured under antiviral pressure

For each drug condition in the resistance selection procedure, two starting concentrations were used, ‘L’ refers to the lower and ‘H’ to the higher one. The frequencies of mutations in the virus cultures were assessed by amplicon-based NGS after Passages 2 and 5. All virus cultures, both selected under monotherapy and combination therapy, acquired mutations after Passage 5 (Table 1). Sanger sequencing was performed on the virus cultures after five passages under antiviral pressure to confirm the mutations that were detected by NGS. Although Sanger sequencing detected most variants with a frequency above 10% (Table 1, footnote c), it failed to detect variants with lower frequencies.

**Table 1.** Frequency of mutations arising following two and five passages under antiviral pressure detected by amplicon-based NGS.

			Frequency (%) ± SD		
Condition	Protein (gene)	Mutation	Passage 2	Passage 5	
**PFA^L^**	DP (*UL30*)	*S724N*	–	85.1 ± 0.13^c^	
		*E798K*	–	2.2 ± 0.00	
		*I890M*	–	4.3 ± 0.09	
		**A910T**	3.1 ± 0.35	–	
**PFA^H^**	DP (*UL30*)	*L802F*	47.7 ± 0.53	51.0 ± 0.09^c^	
		**G943S**	3.3 ± 0.04	–	
		**N962del**	30.4 ± 0.09	94.9 ± 0.05^c^	
		**del C nt 2883**	–	2.6 ± 0.03	
**PTV^L^**	Helicase (*UL5*)	**A236V** ^a^	2.2 ± 0.03	–	
		*K356N*	–	10.8 ± 0.55	
		*K356Q*	–	47.7 ± 0.50^c^	
		**N588T** ^a^	3.3 ± 0.06	–	
		**V752M** ^a^	5.6 ± 0.13	2.5 ± 0.02	
	Primase (*UL52*)	*S364G* ^a^ ^,^ ^b^	10.3 ± 0.39	12.7 ± 0.44^c^	
		**V637A** ^a^	4.9 ± 0.00	4.9 ± 0.03	
		*A899T*	–	5.8 ± 0.08	
		**V976I** ^a^	5.2 ± 0.19	6.2 ± 0.37	
**PTV^H^**	Helicase (*UL5*)	**A236V** ^a^	5.2 ± 0.19	–	
		*K356N*	–	91.8 ± 0.31^c^	
		**V752M** ^a^	7.1 ± 0.49	–	
	Primase (*UL52*)	*S364G* ^a^ ^,^ *^b^*	15.7 ± 0.35	5.8 ± 0.17	
		**V637A** ^a^	3.0 ± 0.28	–	
		**V976I** ^a^	5.5 ± 0.19	–	
**ACV + PTV^L^**	TK (*UL23*)	*V187M*	19.8 ± 0.31	75.9 ± 0.22^c^	
		*del C nts 460-464*	–	14.8 ± 0.11^c^	
		*ins C nts 548-553*	–	4.9 ± 0.19	
	DP (*UL30*)	**D615Y**	1.8 ± 0.02	2.9 ± 0.12	
		A646T	–	2.7 ± 0.17	
		*G841S*	–	25.0 ± 0.12^c^	
	Helicase (*UL5*)	**A236V** ^a^	3.7 ± 0.13	1.1 ± 0.02	
		**V752M** ^a^	6.8 ± 0.12	1.4 ± 0.09	
	Primase (*UL52*)	*S364G* ^a^ ^,^ ^b^	30.1 ± 0.03	80.0 ± 0.60 ^c^	
		**V637A** ^a^	3.5 ± 0.10	–	
		**V976I** ^a^	4.1 ± 0.69	–	
		**R979C**	2.1 ± 0.34	–	
**ACV + PTV^H^**	TK (*UL23*)	*R51W*	3.3 ± 0.09	77.7 ± 2.14 ^c^	
		*A93V*	62.1 ± 1.25	11.8 ± 1.55 ^c^	
		*V187M*	17.9 ± 0.04	–	
		*ins C nts 548–553*	2.9 ± 0.07	1.7 ± 0.43	
	DP (*UL30*)	–	–	–	
	Helicase (*UL5*)	**A236V** ^a^	4.1 ± 0.08	–	
		**L428F**	62.9 ± 0.36	54.4 ± 3.51 ^c^	
	Primase (*UL52*)	**H359Y**	–	3.0 ± 1.54	
		*S364G* ^a^ ^,^ *^b^*	–	15.3 ± 2.16^c^	
**PFA + PTV^L^**	DP (*UL30*)	*S724N*	–	35.1 ± 0.17^c^	
		*L802F*	91.4 ± 0.02	5.5 ± 0.21	
		*R842S*	–	48.0 ± 0.63^c^	
		**T898M**	–	3.7 ± 0.07	
	Helicase (*UL5*)	**V752M** ^a^	–	18.7 ± 0.20^c^	
	Primase (*UL52*)	–	–	–	
**PFA + PTV^H^**	DP (*UL30*)	**V501M**	16.0 ± 0.15	86.8 ± 3.01^c^	
		**L636M**	–	7.5 ± 0.87^c^	
		*L802F*	9.9 ± 0.28	80.1 ± 5.59^c^	
		*I890M*	–	6.2 ± 0.23	
	Helicase (*UL5*)	**V752M** ^a^	19.3 ± 0.02	23.7 ± 1.70^c^	
	Primase (*UL52*)	**A384V**	–	5.3 ± 0.27	
		**V637A** ^a^	1.3 ± 0.03	8.4 ± 8.07^c^	
		**V976I** ^a^	11.4 ± 0.37	14.3 ± 2.29^c^	

Mutations with a frequency <2% at Passages 2 and/or 5 are not included. - : mutation was not detected, bold: novel change, italics: known drug resistance mutation, underlined: known genetic polymorphism (Stranska et al. 2004, Biswas et al. 2008, Chono et al. 2012, Andrei and Snoeck 2013, Collot et al. 2016, Karamitros et al. 2016, Kakiuchi et al. 2017).

a Mutation pre-existing as a minor variant in the parental wild-type strain.

b Mutation previously linked to AMV resistance.

c Mutation also detected by Sanger sequencing of the virus culture at Passage 5.

#### Mutations selected under monotherapy pressure

The mutations arising under PFA or PTV monotherapy pressure were positioned in the DP (PFA^L^ and PFA^H^), helicase (PTV^L^ and PTV^H^), and primase (PTV^L^ and PTV^H^) genes and were detected as heterogeneous populations (Table 1). At Passage 5, three resistance mutations were detected in the DP gene of virus culture PFA^L^, i.e. S724N (85.1%) and minor populations (<5%) of E798K and I890M. In the PFA^H^ virus culture, the L802F resistance mutation (51%) and the novel N962 deletion (94.9%), detected in the DP gene at Passage 5, had emerged fast with frequencies exceeding 30% after Passage 2. As reported in a previous publication, ACV monotherapy pressure induces TK and DP mutations within five passages (Schalkwijk et al. 2024).

Several low-frequency helicase and primase variants were detected in the PTV^L^ and PTV^H^ viral cultures at Passages 2 and 5 (Table 1). Three mutations were detected at frequencies >10% in the helicase (K356N and K356Q) and primase (S364G) genes of the PTV^L^ viral culture at Passage 5, while in the case of PTV^H^, the K356N (91.8%) mutation was the only mutation detected at a frequency >10%. The S364G mutation in the primase has been formerly associated with AMV resistance (Chono et al. 2012), while the K356N/Q mutations in the helicase are associated with both PTV and AMV resistance (Collot et al. 2016).

#### Mutations selected under combinatorial drug pressure

Combination pressure of ACV and PTV slowed down virus growth, as the total days in culture after five passages were higher for the ACV + PTV^L^ (33 days) and ACV + PTV^H^ (26 days) culture conditions than for ACV monotherapy pressure (16–17 days) (Schalkwijk et al. 2024), PTV^L^ (14 days), and PTV^H^ (15 days) (Supplementary Table S1). The total days in culture of PFA^L^ (33 days), PTV^L^, and PTV^H^ were lower than those of PFA + PTV^L^ (42 days) and PFA + PTV^H^ (43 days), whereas the total days in culture of PFA^H^ (47 days) were higher. However, the final drug concentrations used for PFA + PTV^L^ (200 + 0.02 µg/ml) and PFA + PTV^H^ (300 + 0.04 µg/ml) were markedly lower than those for PFA^L^ (800 µg/ml), PFA^H^ (1200 µg/ml), PTV^L^ (0.08 µg/ml), and PTV^H^ (0.16 µg/ml).

Viruses cultured under combinatorial drug pressure accumulated mutations in the TK and/or DP genes at frequencies similar to ACV (Schalkwijk et al. 2024) or PFA monotherapy pressure (Table 1). Deep sequencing of the relevant genes revealed mixed virus populations in all virus cultures selected under combinatorial drug pressure.

In the ACV + PTV^L^ culture, the TK V187M (75.9%) and primase S364G (80%) mutations were predominant at Passage 5 and were already present at a frequency of 20%–30% at Passage 2. Multiple mutations (R51W, A93V, and a C insertion at nucleotides (nts) 548–553) were detected in the TK gene of ACV + PTV^H^ after Passage 5, whereas no mutations were detected in the DP. In addition to the L428F change in the helicase, two changes were detected in the primase gene (H359Y and S364G) of the ACV + PTV^H^ culture, all of which had unknown effects on PTV susceptibility.

Known PFA resistance mutations were detected in the DP gene of viruses emerging under PFA + PTV pressure, but known PTV resistance mutations were not detected in the helicase or primase genes. Along with the S724N (35.1%) and R842S (48%) resistance mutations, the L802F and T898M changes were detected at low frequencies (<10%) in the DP gene of virus culture PFA + PTV^L^. The UL5 V752M was the only change detected in the helicase and primase genes of PFA + PTV^L^ at Passage 5. In the PFA + PTV^H^ culture, two novel DP changes (V501M and L636M) were identified in addition to DP resistance mutations L802F and I890M. The V501M (86.8%) and L802F (80.1%) changes were predominant after five passages and were already detected after Passage 2. The L636M and I890M changes were solely detected after Passage 5 at frequencies <10%. The previously undescribed V752M and V976I changes, detected, respectively, in the helicase and primase gene of PFA + PTV^H^ after Passage 2, did not increase in frequency under further selection pressure.

### Minor UL5 and UL52 variants pre-exist in laboratory KOS strain

The detection of the same low-frequency variants in the helicase and primase genes of the different virus cultures, which did not seem to increase under drug pressure, suggested that they might pre-exist in the laboratory strain. Deep sequencing of the parental KOS strain confirmed the presence of minor variants at a frequency <10% in both the UL5 (A236V, N558T, and V752M) and UL52 genes (S364G, V637A, and V976I) (Table 2). The UL52 S364G mutation has been formerly associated with AMV resistance (Chono et al. 2012), while the other changes have not been described in the literature. Except for the N558T substitution in the helicase, all changes were detected in one or more of the virus cultures after five passages under PTV pressure or under combinatorial drug pressure.

**Table 2.** Frequencies of minor helicase (UL5) and primase (UL52) variants in the laboratory KOS strain before and after five passages under antiviral drug pressure. - : mutation was not detected,

	Frequency (%) ± SD					
Condition	UL5 A236V	UL5 N558T	UL5 V752M	UL52 S364G	UL52 V637A	UL52 V976I
**KOS strain**	3.5 ± 0.20	2.4 ± 0.33	4.6 ± 0.16	8.9 ± 0.14	3.5 ± 0.23	5.9 ± 0.22
**PTV^L^**	–	–	2.5 ± 0.02	12.7 ± 0.44	4.9 ± 0.03	6.2 ± 0.37
**PTV^H^**	–	–	–	5.8 ± 0.17	–	–
**ACV + PTV^L^**	1.1 ± 0.02	–	1.4 ± 0.09	80.0 ± 0.60	–	–
**ACV + PTV^H^**	–	–	–	15.3 ± 2.16	–	–
**PFA + PTV^L^**	–	–	18.7 ± 0.20	–	–	–
**PFA + PTV^H^**	–	–	23.7 ± 1.70	–	8.4 ± 8.07	14.3 ± 2.29

### Genotype of HSV-1 viral clones isolated following antiviral pressure

#### Viral clones selected following monotherapy pressure

Viral clones that were plaque-purified from the virus cultures selected under monotherapy pressure all carried one or more mutations (Table 3). Clones isolated from the PFA^L^ culture all harbored the DP S724N resistance mutation. The five PFA^H^-derived clones bore the novel N962 deletion in the DP gene, with two clones also presenting the L802F mutation. All clones derived from PTV^L^ and PTV^H^ carried mutations at the UL5 K356 locus—K356Q in 4/5 clones derived from PTV^L^ and the K356N mutation was present in the remaining clones. The pre-existing UL52 V637A change was additionally present in a PTV^L^-derived clone harboring the K356Q mutation.

**Table 3.** Genotype of virus clones, plaque-purified after five passages under antiviral pressure.

		Mutations in			
Condition	Number of clones	TK (*UL23*)	DP (*UL30*)	Helicase (*UL5*)	Primase (*UL52*)
**PFA^L^**	5	–	*S724N*	–	–
**PFA^H^**	2	–	*L802F*, **N962del**	–	–
	3	–	**N962del**	–	–
**PTV^L^**	1	–	–	*K356N*	–
	3	–	–	*K356Q*	–
	1	–	–	*K356Q*	**V637A** **^a^**
**PTV^H^**	5	–	–	*K356N*	–
**ACV + PTV^L^**	1	*C ins nts 548–553*	–	–	–
	1	*V187M*	–	–	–
	2	*V187M*	–	–	*S364G^a^*
	1	*V187M*	**Q510H**	–	*S364G^a^*
**ACV + PTV^H^**	2	*R51W*	–	**L428F**	–
	1	*R51W*	–	**L428F**	**H359Y**
	1	*R51W, V187M*	–	**L428F**	**H359Y**
	1	*A93V*	–	**L428F**	**H359Y**
**PFA + PTV^L^**	1	–	*S724N*	–	–
	3	–	*R842S*	–	–
	1	–	*L802F*, **T898M**	–	–
**PFA + PTV^H^**	1	–	*S724N*	–	–
	1	–	**V501M, L636M**	–	–
	1	–	**V501M**, *L802F*	–	–
	1	–	**V501M**, *L802F*	**V752M** **^a^**	**V637A** **^a^**
	1	–	**V501M**, *L802F*	–	**V976I** **^a^**

- : mutation was not detected, bold: novel mutation, italics: known drug resistance mutation (Chono et al. 2012, Andrei and Snoeck 2013, Collot et al. 2016, Karamitros et al. 2016). **^a^** Mutation that waspre-existing as a minor variant in parental laboratory strain KOS.

#### Viral clones selected following combination therapy

ACV resistance mutations were detected in the TK gene of all clones derived from the ACV + PTV^L^ (V187M and C insertion at nts 548–553) and ACV + PTV^H^ (R51W, A93V, and V187M) cultures (Table 3), in concurrence with clones isolated following ACV monotherapy pressure (Schalkwijk et al. 2024). Three ACV + PTV^L^-derived clones harbored the pre-existing S364G primase mutation together with the TK V187M mutation, from which one clone additionally harbored the novel DP Q510H change. All ACV + PTV^H^-derived clones presented the UL5 L428F change with 4/5 clones additionally bearing the UL52 H359Y change.

One PFA + PTV^L^-derived clone bore the DP S724N mutation, three clones harbored the DP R842S mutation, and one clone presented the DP L802F and T898M substitutions. No mutations were detected in the helicase and/or primase genes of PFA + PTV^L^-derived clones. One clone isolated from the PFA + PTV^H^ virus culture presented the S724N mutation in the DP gene. The other four clones harbored the DP V501M change, in combination with either the DP L802F mutation (3 clones) or the DP L636M change (one clone). Two DP V501M + L802F mutant clones, selected under PFA + PTV^H^ pressure, additionally harbored one (UL52 V976I) or two (UL5 V752M and UL52 V637A) pre-existing changes. A clone harboring the DP V501M and UL5 V752M changes could be obtained from the PFA + PTV^H^ virus stock collected after Passage 2.

### Validation of drug resistance evolution under PTV (combination) therapy using plaque-purified virus

**Figure 2.**
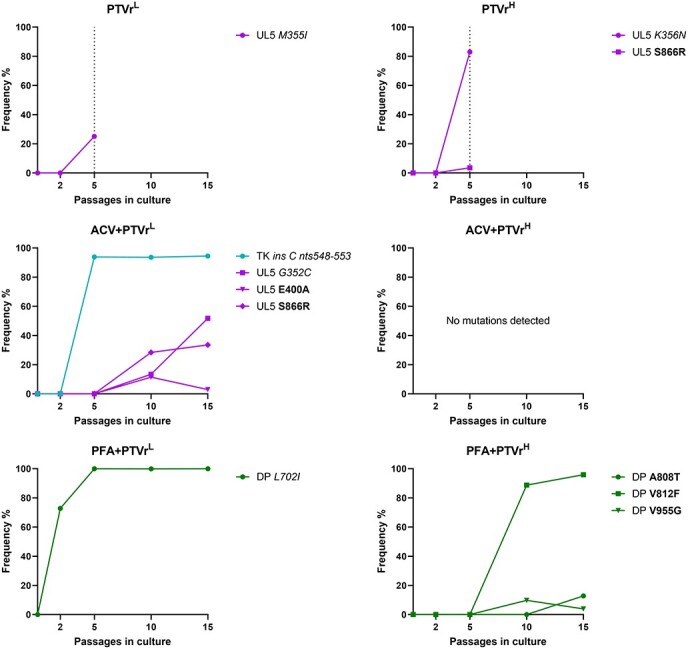
Chronological detection of drug resistance mutations under PTV (combination) pressure using plaque-purified wild-type virus. The frequency (%) of mutations in the virus cultures was defined before and following 2, 5, 10, and 15 passages by deep sequencing of the viral TK (*UL23* gene), DP (*UL30* gene), helicase (*UL5* gene), and primase (*UL52* gene). PTVr^L^ and PTVr^H^ were cultured for five passages (until the dotted line). Bold: previously undescribed change with an unknown effect on drug susceptibility; italics: known drug resistance mutations (Chibo et al. 2004, Saijo et al. 2005, Collot et al. 2016).

The resistance selection procedure under PTV, ACV + PTV, and PFA + PTV pressure was repeated using a plaque-purified KOS strain lacking pre-existing low-frequency subpopulations (Fig. 2 and Supplementary Table S3). Following five passages under PTV pressure, known resistance mutations were acquired in the helicase gene of PTVr^L^ (M355I, 25.1%) and PTVr^H^ (K356N, 82.9%). In addition, a minor population harboring the novel S866R change was detected in the helicase gene of PTVr^H^. Five passages under combinatorial drug pressure induced resistance mutations in the TK gene of ACV + PTVr^L^ (C insertion at nts 548–553, 93.8%) and the DP gene of PFA + PTVr^L^ (L702I, 99.9%), but no mutations were detected in the helicase and/or primase. The virus cultures ACV + PTVr^H^ and PFA + PTVr^H^, from which initial drug concentrations were higher (Supplementary Table S1), acquired no mutations following five passages of the plaque-purified KOS strain.

Additional passaging was performed under combinatorial drug pressure without further increasing the drug concentrations. After Passage 10, the ACV + PTVr^L^ culture acquired three mutations [G352C (13.4%), E400A (11.5%), and S866R (28.4%)] in the helicase, in addition to the TK insertion identified at Passage 5 (Fig. 2 and Supplementary Table S3). While the G352C has been linked to drug resistance (Collot et al. 2016), the E400A and S866R changes had unknown effects on PTV susceptibility. The TK frameshift and helicase mutations G352C, E400A, and S866R were also detected after 15 passages. Virus culture PFA + PTVr^L^, which presented the DP L702I change from Passage 2 onwards, did not acquire further mutations after 15 passages. The PFA + PTVr^H^ culture, which had a wild-type genotype at Passage 5, acquired two novel DP mutations [V812F (88.7%) and V955G (9.8%)] after Passage 10. Following 15 passages, PFA + PTVr^H^ additionally acquired the novel DP A808T (12.8%) substitution, but helicase and/or primase mutations were not detected. The ACV + PTVr^H^ virus culture remained free of mutations after 15 passages under combinatorial drug pressure.

#### Genotype of viral clones isolated following PTV (combination) therapy using plaque-purified virus

Viral clones were isolated from the six virus cultures after five sequential passages of the plaque-purified KOS strain (Table 4). From PTVr^L^, two wild-type and three UL5 M355I mutant clones were isolated, whereas all clones obtained from PTVr^H^ carried the K356N mutation in the helicase gene. The ACV + PTVr^L^-derived clones all presented the C insertion at nts 548–553 of the TK gene. Five clones isolated from PFA + PTVr^L^ all bore the DP L702I mutation. From ACV + PTVr^H^ and PFA + PTVr^H^, only clones with a wild-type genotype were obtained, in agreement with the wild-type genotype of these virus cultures at Passage 5.

**Table 4.** Genotype of virus clones isolated after five passages of PTV (combination) therapy using a plaque-purified virus.

		Mutations in			
Condition	Number of clones	TK (*UL23*)	DP (*UL30*)	Helicase (*UL5*)	Primase (*UL52*)
**PTVr^L^**	2	–	–	–	–
	3	–	–	*M355I*	–
**PTVr^H^**	5	–	–	*K356N*	–
**ACV + PTVr^L^**	5	*ins C nts 548–553*	–	–	–
**ACV + PTVr^H^**	5	–	–	–	–
**PFA + PTVr^L^**	5	–	*L702I*	–	–
**PFA + PTVr^H^**	5	–	–	–	–

- : No mutation detected, bold: novel amino acid change with an unknown effect on drug resistance, italics: known drug resistance mutations (Chibo et al. 2004, Saijo et al. 2005, Collot et al. 2016).

Clones were also plaque-purified from ACV + PTVr^L^ and PFA + PTVr^H^ after Passage 10. ACV + PTVr^L^-derived clones harbored the G352C, E400A, or S866R mutation in the helicase together with the TK C insertion at nts 548–553. All clones isolated from PFA + PTVr^H^ at Passage 10 carried the DP V812F mutation, while a V955G mutant—detected in the virus stock—was not obtained.

### Drug susceptibility of HSV-1 mutants harboring novel mutations

The drug susceptibility of 20 viral clones was evaluated using CPE reduction assays (Table 5). The novel DP mutations V501M, Q510H, V812F, and N962del could be linked to drug resistance. The N962 deletion conferred PFA resistance (3.7-fold) but did not affect susceptibility to the other antivirals tested. The DP Q510H mutation, though only found in combination with changes in the TK and UL52, could be linked to PMEA resistance (three-fold). The V812F mutant clone was resistant to ACV (3.3-fold), PFA (6.2-fold), and PMEA (4.9-fold). The DP V501M mutant and DP V501M + L636M double-mutant were both resistant to ACV, PFA, and PMEA, but the contribution of the L636M change could not be distinguished.

**Table 5.** Resistance profile of HSV-1 viral clones selected under antiviral pressure.

	Mutations in				MFR							
Condition	*UL23*	*UL30*	*UL5*	*UL52*	ACV	GCV	TFT	PFA	PMEA	CDV	PTV	AMV
PFA^H^		**N962del**			0.9	0.9	0.5	**3.7**	0.8	1.5	0.7	0.5
PTV^L^			*K356Q*		0.8	1.0	0.6	0.7	0.5	0.9	**33**	**31**
			*K356Q*	**V637A**	0.5	1.3	0.5	0.6	0.6	0.9	**37**	**35**
PTV^H^			*K356N*		0.9	1.2	0.5	0.8	0.6	1.0	**≥78**	**≥482**
ACV + PTV^L^	*V187M*			*S364G*	**20**	**21**	0.5	0.7	1.4	1.3	1.6	**2.0**
	*V187M*	**Q510H**		*S364G*	**46**	**5.5**	0.4	0.9	**3.0**	1.1	1.5	**2.2**
ACV + PTV^H^	*R51W*		**L428F**		**251**	**558**	0.9	0.8	0.9	0.9	1.2	1.0
	*R51W*		**L428F**	**H359Y**	**≥251**	**≥770**	1.3	0.9	1.5	1.0	1.7	1.1
PFA + PTV^H^		**V501M**	**V752M**		**2.9**	1.6	0.9	**3.2**	**2.3**	1.5	1.1	1.1
		**V501M, L636M**			**3.2**	1.3	0.7	**2.8**	**2.4**	1.8	1.2	0.9
		**V501M**, *L802F*			**3.2**	1.1	0.7	**3.8**	**2.6**	1.6	1.0	0.9
		**V501M**, *L802F*	**V752M**	**V637A**	**4.7**	**2.4**	0.7	**3.7**	**3.6**	1.9	1.4	1.0
		**V501M**, *L802F*		**V976I**	**3.2**	1.9	1.0	**3.6**	**2.6**	1.8	0.4	0.6
PTVr^L^			*M355I*		0.9	0.9	1.1	1.0	0.7	0.5	**6.8**	**7.0**
ACV + PTVr^L^	*ins C nts 548–553*				**≥286**	**832**	0.5	0.8	1.1	0.4	0.5	0.5
	*ins C nts 548–553*		*G352C*		**≥93**	**≥269**	0.7	0.9	1.3	0.8	**29**	**13**
	*ins C nts 548–553*		**E400A**		**≥93**	**≥269**	0.7	0.6	1.2	0.8	**4.5**	**2.1**
	*ins C nts 548–553*		**S866R**		**≥93**	**≥269**	0.6	0.6	0.9	0.7	**6.6**	1.8
PFA + PTVr^L^		*L702I*			**4.0**	0.6	0.9	**4.6**	**4.3**	1.6	1.6	1.0
PFA + PTVr^H^		**V812F**			**3.3**	0.9	0.7	**6.2**	**4.9**	1.5	0.8	0.5

Novel changes are marked bold. The mean EC_50_ (50% effective concentration) and fold resistance (EC_50_ mutant/EC_50_ wild-type) were determined by CPE reduction assays (≥3 independent experiments). A MFR value ≥2 was considered resistant and is indicated in bold. Mean EC_50_ of wild-type: ACV (0.09 µg/ml ± 0.07), GCV (0.006 µg/ml ± 0.005), TFT (0.98 µg/ml ± 0.2), PFA (38.7 µg/ml ± 2.6), PMEA (24.1 µg/ml ± 10.6), CDV (0.6 µg/ml ± 0.3), PTV (0.02 µg/ml ± 0.008), AMV (0.02 µg/ml ± 0.008).

Abbreviation: MFR, mean fold resistance.

**Figure 3.**
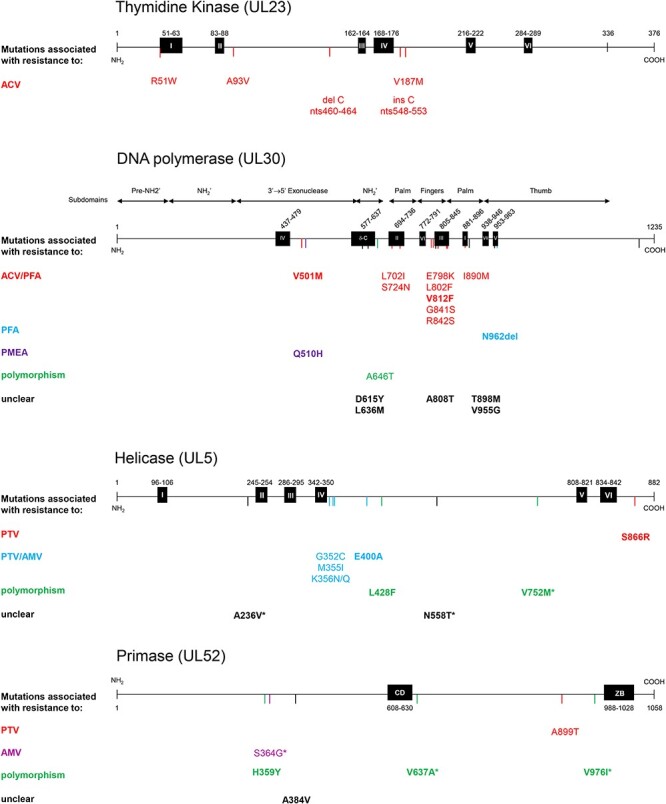
Mutation map of the HSV-1 TK (*UL23*), DP (*UL30*), helicase (*UL5*), and primase (*UL52*) mutations identified in this study. Conserved sites are indicated by black boxes. Alterations that were not detected in a viral clone or with a frequency of >2% at Passages 5, 10, and/or 15 were excluded. Bold: previously undescribed changes. * Pre-existing as minor variant in the parental KOS strain.

Both the K356N and K356Q mutations in the helicase were associated with high-level (>30-fold) PTV and AMV resistance. Resistance to PTV and AMV was also observed in clones bearing the helicase mutations G352C (29- and 13-fold), M355I (6.8- and 7.0-fold), and E400A (4.5- and 2.1-fold). The UL5 S866R mutant clone was resistant to PTV (6.6-fold) but not to AMV (1.8-fold), whereas two clones harboring the S364G mutation in the primase gene revealed low-level AMV resistance (2.0- and 2.2-fold) but remained susceptible to PTV (1.6- and 1.5-fold). Clones harboring the UL5 L428F change individually or in combination with the UL52 H359Y change were susceptible to PTV or AMV, pointing toward natural genetic polymorphisms. Furthermore, clones presenting the V752M change in the helicase and/or the V637A and V976I changes in the primase remained susceptible to PTV and AMV, confirming that these pre-existing substitutions are linked to genetic polymorphisms An overview of all mutations identified in this study and their associated phenotype is available in Fig. 3.

## Discussion

In this study, we described the effects of PTV combination therapy on HSV-1 inhibition and drug resistance emergence. Combining drugs that do not share resistance patterns is a powerful approach to increase the genetic barrier to resistance, i.e. the number and types of mutations in an antiviral target necessary to become resistant to the therapy (Nijhuis et al. 2009, Irwin et al. 2016). Since PTV has a mechanism of action dissimilar to ACV and PFA, their combination seems to be promising as an antiviral strategy.

Two conditions have to be met for a virus strain to emerge under antiviral pressure. First, the virus strain must be resistant to the drug (combination), and second, it has to be able to replicate in sufficient quantities (Nijhuis et al. 2009). Resistance to one of the drugs used in combination therapy does not necessarily abolish the efficacy of the therapy (Campbell et al. 1993, Hobden et al. 2011). Therefore, resistance to both drugs is likely needed for a virus to escape combinatorial drug pressure. In this study, selection pressure with the individual antivirals induced drug resistance within five passages. Combined selection pressure of ACV and PTV resulted in ACV resistance in three out of four virus cultures, from which only one acquired resistance to both selection drugs, albeit with a notable delay. PFA and PTV combination pressure resulted in PFA resistance but prevented the emergence of PTV resistance in all four virus cultures. This suggests that there is a higher genetic barrier for PFA + PTV combination therapy than for ACV + PTV combination therapy. In this study, resistance selection was continued for 5–15 passages and resistance to both selection drugs might still emerge upon further passaging.

Drug resistance mutations can reduce the replicative capacity of a virus, especially when located in proteins vital for replication. The viral DP, helicase, and primase proteins are all essential for viral replication, whereas TK activity is only essential for replication in nondividing cells that lack sufficient cellular TK activity, such as neurons (Boehmer and Lehman 1997). TK-defective HSV-1 strains grow at rates and titers equal to wild-type in cell culture but show impaired reactivation and decreased pathogenicity in mice (Omura et al. 2017). HSV-1 DP mutants generally exhibit reduced replication capacities in cell culture and exhibit near wild-type or attenuated neurovirulence in mice (Andrei et al. 2007, Zarrouk et al. 2021). Only a few PTV or AMV resistance mutations have been identified in the primase, and their effects on replication capacity have not been extensively studied (Biswas et al. 2008, Sato et al. 2021). Most resistance mutations in the helicase, including the G352C mutation, show reduced growth in cell culture and reduced pathogenicity in mice (Field and Biswas 2011, Sato et al. 2021). Exceptions are mutations at the K356 locus (K356N and K356Q). The K356Q mutation shows increased replication capacity in tissue culture (Biswas, et al., 2007a). The K356N mutation confers the highest level of PTV resistance of all helicase mutations described to date, is frequently selected under antiviral pressure *in vitro*, and shows near wild-type growth properties (Biswas and Field 2008).

The acquisition of drug resistance mutations in multiple essential genes might severely impair viral replication capacity. Virus strains harboring resistance mutations in both the TK and helicase genes were isolated from ACV + PTVr^L^. These strains first acquired a frameshift mutation in the TK (C insertion at nts 548–553), while mutations in the helicase were acquired upon further passaging. Interestingly, the helicase mutations were not positioned at the K356 locus but were dispersed throughout the helicase (G352C, E400A, and S866R). In this study, only one clone, selected under ACV + PTV combination therapy, harbored mutations in the TK (V187M), DP (Q510H), and primase (S364G) simultaneously. Further research should assess the replication capacity of viruses harboring drug resistance mutations in multiple genes.

Previous research showed that mutations conferring resistance to helicase–primase inhibitors can pre-exist at low frequencies in HSV-1 laboratory strains and in clinical isolates from therapy-naïve individuals (Biswas, et al., 2007b, Sukla et al. 2010). We detected multiple minor helicase and primase variants in our KOS laboratory strain prior to the selection procedure by deep sequencing, including the primase S364G mutation. Mutants harboring the S364G change have previously shown ambiguous results with not all mutants displaying AMV resistance (Chono et al. 2012, Sato et al. 2021), in agreement with the borderline (2.0- to 2.2-fold) resistance observed in this study. A few of the pre-existing variants increased in frequency following PTV (combination) pressure, but this was inconsistent among the different conditions. In ACV + PTV^L^, the primase S364G mutation was present at an 80% frequency, likely due to its co-occurrence in the same virus backbone as the TK V187M mutation. Whether ACV/PTV or PFA/PTV (multi)drug-resistant variants also pre-exist in laboratory strains or clinical isolates should be investigated in future work.

In this study, an amplicon-based approach was used to sequence the *UL23, UL30, UL5*, and *UL52* genes, where mutations conferring drug resistance locate. This resulted in high coverage (>200.000 reads per amplicon), enabling the detection variants with a sensitivity of 1%. Most studies exploring deep sequencing for resistance screening have utilized a similar approach (Fujii et al. 2018, Mercier-Darty et al. 2018, 2019, Ávila-Ríos et al. 2020). To date, mutations conferring resistance to ACV, PFA, or PTV have not been identified outside the *UL23, UL30, UL5*, and *UL52* genes, making it unlikely that resistance mutations were missed by our amplicon-based strategy. Whole-genome sequencing may be utilized in future research to identify the presence of potential compensatory mutations outside these target regions that may have been co-selected during the experiments. Additional deep sequencing of the helicase–primase complex accessory protein (*UL8* gene) of the KOS strain before and after 15 passages under combinatory drug pressure (to screen for potential PTV or AMV resistance mutations) did not reveal the existence of mutations (data not shown).

Although the results presented here are promising, the extent to which combination therapy is effective in treating and preventing (drug-resistant) HSV-1 infections *in vivo* remains to be explored. The development of a combination therapy must consider potential drug interactions and differences in the absorption, metabolism, and distribution of the drugs that may alter its combined efficacy and applicability (Pirrone et al. 2011, Greeley et al. 2020). However, combining two drugs may allow the use of lower drug concentrations without decreasing the treatment efficacy and could therefore reduce toxic side effects of the drugs, including nephrotoxicity and neurotoxicity (Leowattana 2019, Brandariz-Nuñez et al. 2021). A better understanding of the *in vivo* efficacy and safety is needed if one wants to implement combination therapy in the clinic. The implementation of a multidrug regimen would be particularly beneficial for immunocompromised individuals, who often experience severe HSV-1 infections prone to becoming resistant.

## Supplementary Material

veae101_Supp

## Data Availability

Raw sequencing data were submitted to the GenBank SRA database under BioProject PRJNA1119278.
