# Supplementary material for: Combined use of pritelivir with acyclovir or foscarnet suppresses evolution of HSV-1 drug resistance
Source: Virus Evol. 2024 Nov 23;10(1):veae101. doi: 10.1093/ve/veae101 (PMC11665824; doi:10.1093/ve/veae101)

Supplementary material

**Table S1.** Drug concentrations used during the resistance selection procedure.

| **Condition** | **Start concentration** (µg/mL) | **Concentration at passage 5** (µg/mL) | **Total days in culture** | |
| --- | --- | --- | --- | --- |
|  |  |  | **Passage 5** | **Passage 15** |
| **PFA^L^** | 50 | 800 | 33 | NP |
| **PFA^H^** | 75 | 1200 | 47 | NP |
| **PTV^L^** | 0.005 | 0.08 | 14 | NP |
| **PTV^H^** | 0.01 | 0.16 | 15 | NP |
| **ACV+PTV^L^** | 0.05 + 0.005 | 0.8 + 0.08 | 33 | NP |
| **ACV+PTV^H^** | 0.1 + 0.01 | 1.6 + 0.16 | 26 | NP |
| **PFA+PTV^L^** | 50 + 0.005 | 200 + 0.02 | 42 | NP |
| **PFA+PTV^H^** | 75 + 0.01 | 300 + 0.04 | 43 | NP |
| **PTVr^L^** | 0.005 | 0.08 | 16 | NP |
| **PTVr^H^** | 0.01 | 0.16 | 19 | NP |
| **ACV+PTVr^L^** | 0.05 + 0.005 | 0.2 + 0.02 | 37 | 74 |
| **ACV+PTVr^H^** | 0.1 + 0.01 | 0.2 + 0.02 | 60 | 159 |
| **PFA+PTVr^L^** | 50 + 0.005 | 200 + 0.02 | 46 | 106 |
| **PFA+PTVr^H^** | 75 + 0.01 | 150 + 0.02 | 64 | 129 |

NP: not performed. Abbreviations: ACV, acyclovir; PFA, foscarnet; PTV, pritelivir.

**Table S2.** List of primers used for amplicon based next-generation sequencing.

| **Gene** | **Protein** | **Amplified region** | **Forward primer** | **Reverse primer** |
| --- | --- | --- | --- | --- |
| UL5 | Helicase | c.23-2649 | GCCAGCTAGACGGACAGAAA | GGGGTGTGCTGGATAACAAC |
| UL23 | Thymidine Kinase | c.1-1128 | TAACAGCGTCAACAGCGTGCC | GAACAAACGACCCAACACCCG |
| UL30 | DNA polymerase | c.900-3427 | CACCCGGTTCATCCTGGACAA | CCAGCTCGGACACCAGCAGC |
| UL52 | Primase | c.1-3177 | CCACATATAGCCCCGAGAAG | GTAGCAGATATGGGCGTGGT |

**Table S3.** Frequency of mutations arising after 2, 5, 10, and 15 passages under PTV (combinatorial pressure) using plaque-purified wild-type virus, detected by amplicon-based next-generation sequencing.

| **Condition** | **Gene** | **Mutation** | **Frequency % ± SD** | | | |  |
| --- | --- | --- | --- | --- | --- | --- | --- |
|  |  |  | **Passage 0** | **Passage 2** | **Passage 5** | **Passage 10** | **Passage 15** |
| PTVr^L^ | UL5 | *M355I* | - | - | 25.1 ± 0.54 * | NP | NP |
|  | UL52 | - | - | - | - | NP | NP |
| PTVr^H^ | UL5 | *K356N* | - | - | 82.9 ± 0.24 * | NP | NP |
|  |  | **S866R***^§^* | - | - | 3.6 ± 0.32 | NP | NP |
|  | UL52 | - | - | - | - | NP | NP |
| ACV+PTVr^L^ | UL23 | *Ins C nts 548-553* | - | - | 93.8 ± 0.14 * | 93.6 ± 0.71 * | 94.5 ± 0.43 * |
|  | UL30 | - | - | - | - | - | - |
|  | UL5 | *G352C* | - | - | - | 13.4 ± 0.14 * | 51.7 ± 0.26 * |
|  |  | **E400A***^§^* | - | - | - | 11.5 ± 0.07 * | 3.0 ± 0.15 |
|  |  | **S866R***^§^* | - | - | - | 28.4 ± 0.01 * | 33.6 ± 0.71 * |
|  | UL52 | - | - | - | - | - | - |
| ACV+PTVr^H^ | UL23 | - | - | - | - | - | - |
|  | UL30 | - | - | - | - | - | - |
|  | UL5 | - | - | - | - | - | - |
|  | UL52 | - | - | - | - | - | - |
| PFA+PTVr^L^ | UL30 | *L702I* | - | 72.8 ± 0.47 | 99.9 ± 0.02 * | 99.9 ± 0.02 * | 100 ± 0.01 * |
|  | UL5 | - | - | - | - | - | - |
|  | UL52 | - | - | - | - | - | - |
| PFA+PTVr^H^ | UL30 | **A808T** | - | - | - | - | 12.8 ± 0.37 |
|  |  | **V812F***^§^* | - | - | - | 88.7 ± 0.05 * | 95.9 ± 0.40 * |
|  |  | **V955G** | - | - | - | 9.8 ± 0.14 * | 3.9 ± 0.12 |
|  | UL5 | - | - | - | - | - | - |
|  | UL52 | - | - | - | - | - | - |
| Frequencies detected by next-generation sequencing are marked in the following colors: 0-10%, 10-20%, 20-40%, 40-60%, 60-80%, 80-100%. **Bold**: novel amino acid change with unknown effect on drug susceptibility, *italics*: known drug-resistance mutations (Chibo et al., 2004; Collot et al., 2016; Saijo et al., 2005). * Mutation was also detected by Sanger sequencing of the virus culture at passage 5, 10 and/or 15, *^§^* mutation linked to drug resistance in this study. NP: not performed. | | | | | | | |


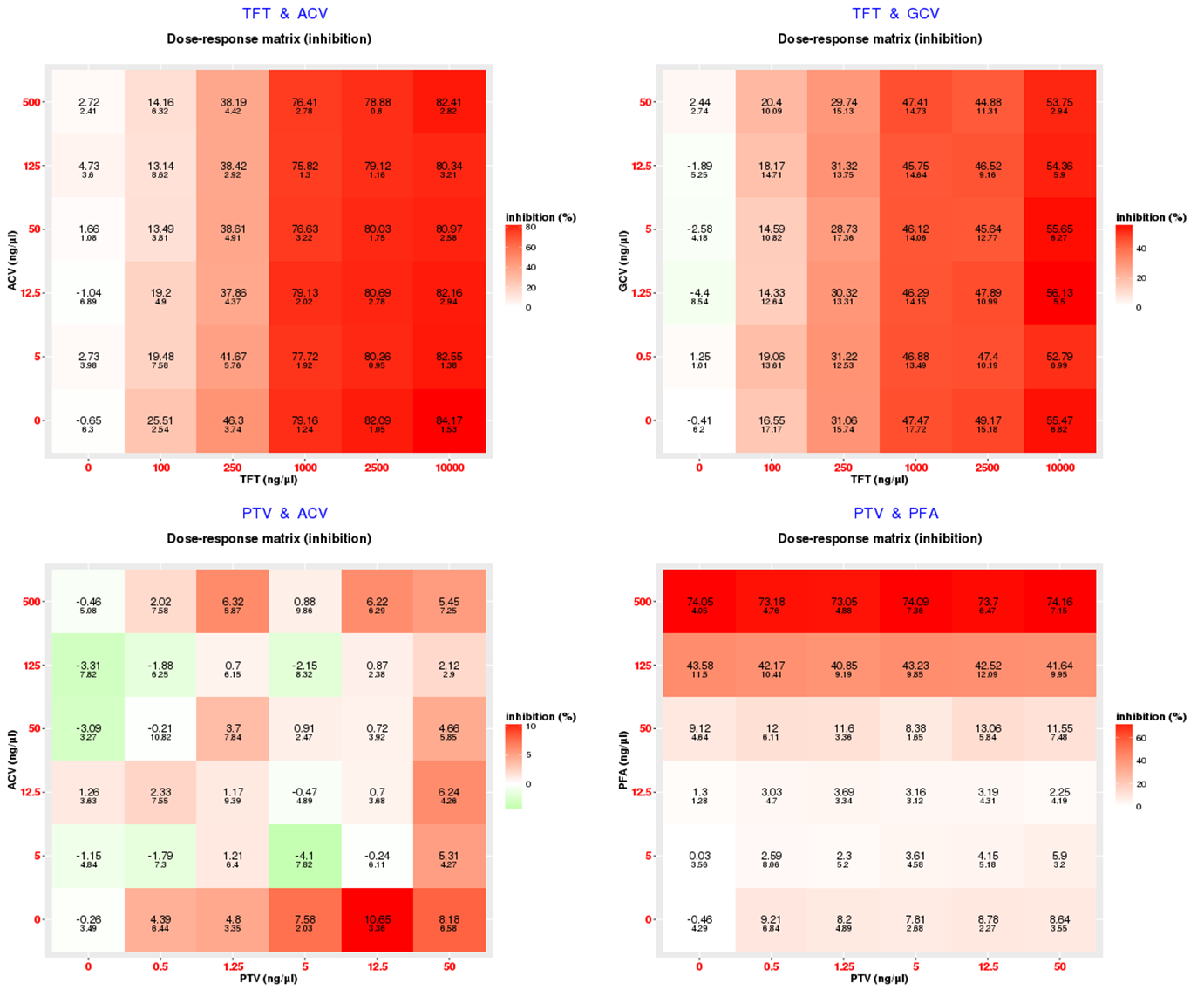
**Fig. S1.** Cytotoxic activity of antiviral compounds in combination. HEL fibroblasts were exposed to various concentrations of one or two antiviral compounds. Cell counts were determined with a Z1 Coulter Counter following three days of incubation (n=4). Overall synergy scores were calculated for acyclovir (ACV) and pritelivir (PTV) (-4.205), and foscarnet (PFA) and PTV (-2.633) using the Zero Interaction Potency (ZIP) model. Synergy scores ˂ -10 suggest antagonistic interactions, scores between -10 and 10 suggest additive effects, and scores >10 indicate synergistic effects. The presented dose-response matrices were generated with SynergyFinder 2.0. The % inhibition indicate the reduction of cell count relative to untreated cells


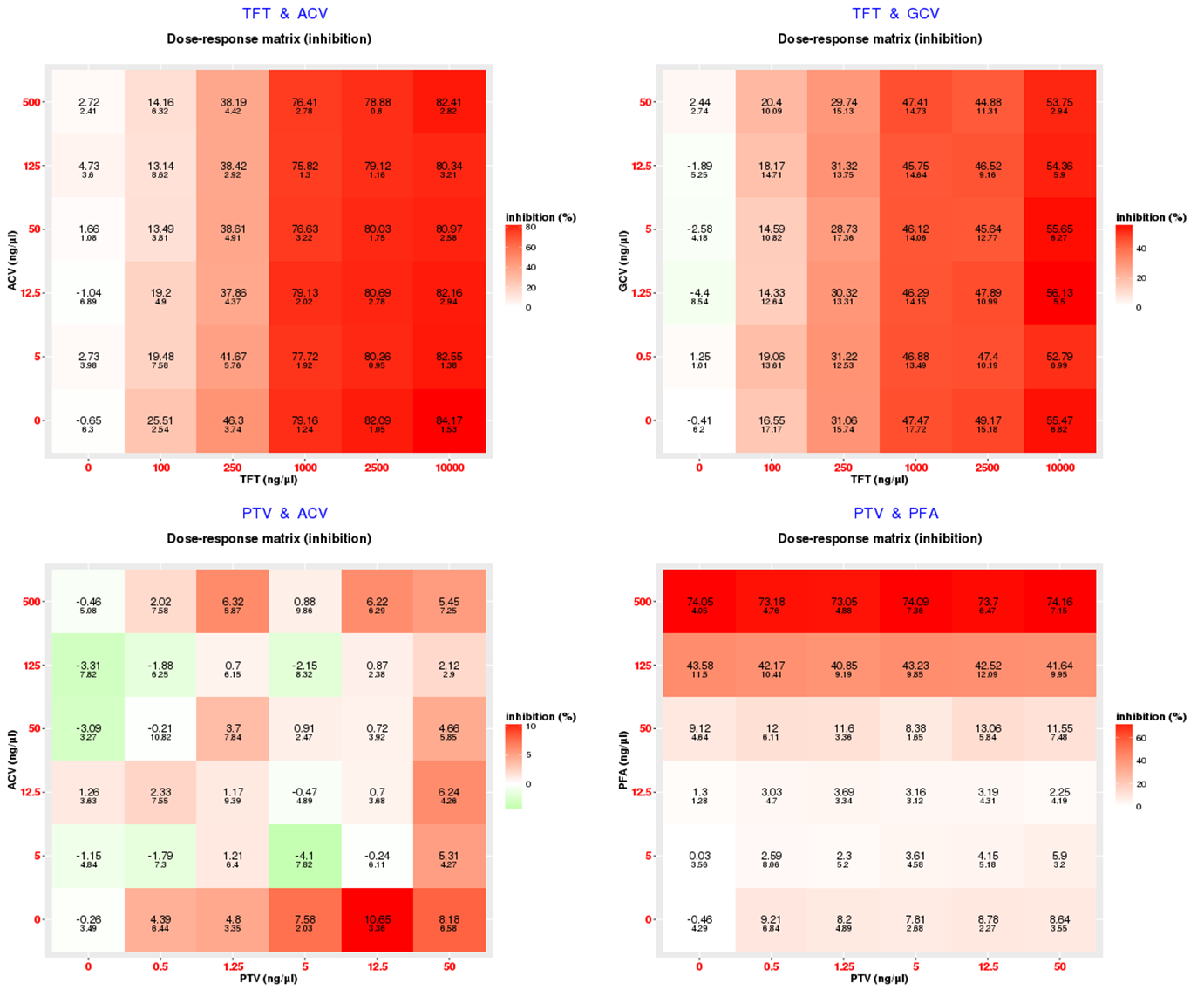

Supplement: veae101_Supp [file veae101_supp.zip › Supplementary material - PTV paper.docx]
